# Integrative Metabolomic and Physiological Responses of *Citrus sinensis* to Soil Management in a Semi-Arid Orchard

**DOI:** 10.3390/plants15030386

**Published:** 2026-01-27

**Authors:** Carlos Giménez-Valero, Dámaris Núñez-Gómez, Pilar Legua, Juan José Martínez-Nicolás, Vicente Lidón, Pablo Melgarejo

**Affiliations:** Plant Production and Microbiology Department, Miguel Hernandez University, Ctra. Beniel km 3.2, 03312 Orihuela, Spain

**Keywords:** *Citrus sinensis*, metabolomics, soil management, mulching, zeolite, fruit quality, semi-arid agriculture

## Abstract

The coordination between carbon and nitrogen metabolism is central to plant adaptation to water-limited environments. This study investigated how soil management practices modulate the metabolic and physiological performance of *Citrus sinensis* trees cultivated under semi-arid conditions. Six field treatments combining weed-control netting, subsurface drainage, and zeolite amendment were evaluated for their effects on vegetative growth, yield, and fruit metabolome. Using ^1^H-NMR spectroscopy, 23 metabolites in peel and 21 in juice were identified and quantified, revealing that sugars, organic acids, and amino acids were the most responsive compound classes. Multivariate analyses (PCA, PLS-DA) showed distinct metabolic fingerprints associated with each soil management regime. Treatments integrating netting and zeolite (*T4*) induced a coordinated reprogramming of carbon and nitrogen metabolism, characterized by altered levels of glucose, fructose, citrate, and proline. These changes suggest enhanced osmotic regulation and tricarboxylic acid cycle activity, supporting improved water-use efficiency and physiological stability under semi-arid stress. The results demonstrate that soil management directly influences fruit metabolic homeostasis, linking environmental modulation of root-zone conditions with whole-plant biochemical adjustment. This integrative metabolomic approach provides mechanistic insight into how soil–plant interactions shape the metabolic resilience of citrus under water-limited field environments.

## 1. Introduction

Water scarcity is one of the main challenges to global food security, with a particular impact on production systems located in arid and semi-arid regions [1]. Climate change is intensifying drought events, reducing the availability of freshwater, and increasing the frequency of heat waves, creating a scenario of greater water stress for crops [2]. In the Mediterranean region, characterized by mild winters, hot summers, and irregular rainfall, the water balance is often negative for much of the year, limiting the productive potential of numerous woody crops [3]. Under these conditions, the efficient use of irrigation water is not only an agronomic necessity but also a requirement for the sustainability of agroecosystems and farm profitability [4].

Sweet orange cultivation (*Citrus sinensis* L. Osbeck) is strategic for the Mediterranean economy. Spain is the leading producer and exporter of citrus fruits in the European Union and one of the largest in the world [5]. National orange production exceeds 2.7 million tons annually, concentrated almost exclusively in the Valencian Community and Andalusia, which together account for more than 90% of production [5]. This economic importance is threatened by increasing pressure on water resources and the need to maintain high fruit quality standards for national and international markets [6]. Faced with this challenge, it is essential to develop soil and irrigation management strategies that improve water use efficiency without compromising fruit yield or organoleptic quality [4,6].

Various agronomic practices have been proposed to mitigate the effects of water deficit, including the use of weed control nets or mulches, which reduce soil evaporation and improve the root microclimate; the installation of underground drainage systems, which allow managing the water table and preventing flooding; and the incorporation of mineral amendments such as zeolite, which can increase cation exchange capacity and water retention [7,8,9]. While the individual effects of these practices on citrus growth have been reported, there is little information on their combined impact on vegetative development, production, and fruit biochemical composition [9,10]. This type of information is crucial for guiding integrated management programs that balance productivity and sustainability.

In recent years, omics sciences have revolutionized the way we study crop responses to stress. Metabolomics, in particular, offers a comprehensive snapshot of the metabolites present in a tissue at a given time, providing an integrated view of biochemical and physiological processes [11]. In citrus, metabolomics has been applied to study responses to pathogens, the variability of bioactive compounds, and changes during fruit ripening [10,12]. However, studies focusing on the comparative metabolomic profile of peel and juice in response to different agronomic practices are still very limited. Furthermore, it is also essential to consider that various environmental factors can significantly influence the accumulation of metabolites in the peel and juice of citrus fruits. Among the most relevant are temperature, solar radiation, and water availability, as these modulate key physiological processes such as photosynthesis, respiration, and the biosynthesis of secondary compounds. Understanding how these management strategies modulate the accumulation of amino acids, sugars, and organic acids could open new opportunities to improve the nutritional and sensory quality of the fruit and guide more sustainable production programs.

Based on the above, this study aimed to evaluate the impact of different soil management strategies—weed control, subsurface drainage, and zeolite application—on vegetative growth, yield, and the metabolomic profile of peel and juice in the sweet orange tree ‘Navelina’. The working hypothesis is that these practices, individually or in combination, improve water and nutrient availability, promote greater vegetative growth, and modulate the biosynthesis of key metabolites, thereby contributing to increased water efficiency, fruit quality, and crop sustainability under water-scarce conditions.

## 2. Materials and Methods

### 2.1. Plant Material and Experimental Design

The study was carried out with sweet orange trees (*Citrus sinensis* L. Osbeck) of the ‘Navelina’ variety, grafted onto a *Citrus macrophylla* Wester rootstock, selected for its widespread use for growing citrus in the Mediterranean region due to its vigor and adaptation to calcareous soils. The seedlings, 21 months old at the start of the trial, were planted in an experimental plot located at the Escuela Politécnica Superior de Orihuela (EPSO), Miguel Hernández University of Elche, in southeastern Spain (38°4′7.70″ N; 0°59′1.11″ W).

The experimental design consisted of six treatments (*T0*–*T5*), defined by the presence or absence of weed control mesh, subsurface drainage, and soil amendments (Table 1 and Supplementary Material). Each treatment was implemented in a plot composed of 21 trees, distributed in three rows of seven specimens, with a planting frame of 6 m × 4 m. All plots were managed following conventional agricultural practices for citrus in the region.

All treatments were irrigated using localized drip irrigation, using two 16 mm diameter low-density polyethylene lines (Azud tub PE model, AZUD S.A., Murcia, Spain). Each tree was provided with four 2 L h^−1^ self-compensating drippers (PC dripper flat outlet, Netafim), two per line and spaced 0.8 m apart. Irrigation scheduling was adjusted based on data from humidity and salinity probes (Sentek Drill and Drop Triscan, Sentek Technologies, Stepney, SA, Australia) installed in all plots, with sensors at 10 cm depth intervals.

The experiment lasted 16 months. After spring flowering, fruit set, and the end of the characteristic physiological drop of the crop in the region [13], fruit counts were carried out per tree in all treatments. The marked heterogeneity in fruit load observed between these trees indicated that trees with higher production could not maintain vegetative development comparable to those with lower loads [14,15,16,17,18]. Since the main objective of this stage was to evaluate the effect of management systems on vegetative growth, uniform thinning was carried out to homogenize the productive load: excess fruit was removed from all trees that exceeded 10 units, ultimately establishing a standard load of 10 fruits/tree until the end of the campaign and its harvest.

### 2.2. Agronomic Characteristics of the Soil

The soil profile was classified as clayey-loam in texture, according to the USDA classification [19], with an approximate composition of 34.15% sand, 33.35% clay, and 32.50% silt, which categorizes it as a medium-textured soil. The pH was slightly alkaline (pH = 8.00), determined according to the ISO 10390 standard [20]. For measurement, a soil–water suspension (1:5) was prepared, stirred, and allowed to equilibrate before being analyzed using a calibrated benchtop pH meter (HI 3220 + mV/°C, Hanna^®^ Instruments, Éibar, Gipuzkoa, Spain) equipped with a glass electrode.

The electrical conductivity (EC) was 2.36 mS cm^−1^, measured in a soil–water suspension (1:2) according to standard UNE-EN 13038 [21], using a conductivity meter with automatic temperature compensation. The apparent density was 1.42 g cm^−3^, and the C/N ratio reached 11.07, reflecting a moderate potential for mineralization of the organic matter.

Chemical analysis showed elevated concentrations of sulfates (835.20 mg L^−1^) and chlorides (184.60 mg L^−1^), along with significant levels of sodium (159.85 mg L^−1^) and calcium (288.00 mg L^−1^). Determinations were made from soil extracts (10 g of dry and sieved sample + 20 mL of deionized water), shaken and filtered to remove particles. Anions were separated by ion chromatography (DIONEX ICS-1000, Thermo Fisher Scientific, Waltham, MA, USA) with a conductivity detector and an IonPac AS11-HC column (4 × 250 mm) with an AG11-HC guard column (4 × 50 mm) (Thermo Fisher Scientific Inc., Waltham, MA, USA). Elution was isocratic with 30 mM NaOH at 1.0 mL min^−1^ and 30 °C, using an AMMS-ICE suppressor (Thermo Fisher Scientific Inc., Waltham, MA, USA). The injection volume was 25 μL, and data were analyzed with Chromeleon 7.3 (Thermo Fisher Scientific, Waltham, MA, USA). This procedure follows standardized methodologies for environmental and agronomic analysis [22].

The total limestone content was very high (56.90%), determined using a Bernard calcimeter, based on the release of CO_2_ by reaction with HCl and volumetric measurement [23,24]. Organic matter was estimated by loss on ignition (LOI), drying the samples at 105 °C and subsequently calcining them at 550 °C. The value obtained (1.43%) suggests limited biological activity and a lower water and nutrient retention capacity compared to soils with higher carbon content.

All analyses were carried out in a laboratory accredited by the National Accreditation Entity (ENAC), complying with the UNE-EN ISO/IEC 17025 standard [25], guaranteeing the reliability of the results.

### 2.3. Local Weather Conditions

The experiment was carried out in Orihuela (Alicante, Spain), under a dry, semi-arid, and subtropical Mediterranean climate, according to the Köppen–Geiger classification [26]. This region has high sunshine (≈3000 h year^−1^) and an average annual temperature slightly above 20 °C, favorable conditions for species adapted to warm and dry environments [27]. The annual temperature oscillation varies between ≈5 °C in winter and 32 °C in summer, with summer periods of more than 3 months where peaks > 35 °C have been recorded in recent years [28].

Winters are moderate, with average temperatures close to 20 °C and minimum temperatures that can drop to 5 °C, allowing citrus fruit to develop without the risk of severe frost damage [29]. Rainfall is scarce and irregular, concentrated in autumn (≈34 L m^−2^ in October) [30]. This, combined with high potential evapotranspiration, generates a marked water deficit that influences irrigation planning [31] and justifies the implementation of efficient water management strategies [32].

### 2.4. Methodology Used for Physical Measurements and Fruit Counting

Twenty-one trees were evaluated per treatment, except for *T5*, which represented 28. The following were measured:

- **Height:** from the collar to the highest branch, using a Medid tape measure (5–19 mm, Medid S.A., Barcelona, Spain).
- **Crown diameter**: average of four measurements (four axes in a cross), representing the average width of the tree.
- **Trunk diameter:** measured 2 cm above the grafting point, using a Mitutoyo ABS Digimatic CD-15APX-150 mm digital caliper (Mitutoyo Corporation, Kawasaki, Japan). The measurement position was permanently marked to ensure comparability over time.

Fruit counting was performed by direct visual inspection, dividing each tree into two sections and counting all the units. This methodology allowed for an accurate count, suitable for small young trees.

### 2.5. Metabolomic Profile of Juice and Peel

Metabolomic characterization was performed using proton nuclear magnetic resonance spectroscopy (^1^H-NMR). Fruits were collected from the 21 trees in each treatment, weighed, and placed in 1 m^3^ macroboxes per block. Fifteen representative fruits per treatment, free of damage and disease symptoms, were selected. After washing, physical measurements were taken, the juice was extracted, and the peels were chopped and temporarily stored at approximately 4 °C.

Subsequently, both juice and peels were stored at −80 °C until freeze-drying (Christ Alpha 2-4 LSCplus, Martin Christ, Osterode am Harz, Germany). Freeze-dried peels were ground (TSM6A013, Taurus, Oliana, Spain) until a homogeneous mixture was obtained. Three biological replicates were analyzed per treatment, each replicate consisting of a composite sample of five fruits, resulting in a total of 15 fruits represented per treatment in the metabolomic analysis.

The extraction of polar and semipolar metabolites followed the protocol described by Van der Sar et al. [33]. Briefly, 50 mg of lyophilized sample was extracted with 1200 μL of MeOH:H_2_O (1:1), followed by shaking, sonication (3 × 1 min), cooling at 4 °C for 30 min, and centrifugation (11,000× *g*, 20 min). The supernatant was evaporated using a SpeedVac at ≤30 °C (Thermo Fisher Scientific Inc., Waltham, MA, USA), and the residue was reconstituted in 800 μL of 100 mM KH_2_PO_4_ buffer prepared in D_2_O containing 0.58 mM TSP. The solution was filtered through a 0.45 μm nylon filter (Merck KGaA, Darmstadt, Germany), and 600 μL was transferred to 5 mm NMR tubes (Wilmad-LabGlass, Vineland, NJ, USA) for analysis.

^1^H-NMR spectra were acquired using a 500 MHz Bruker spectrometer (Bruker Biospin, Rheinstetten, Germany) equipped with a 5 mm N_2_ broadband Prodigy BBO cryogenic probe (Bruker BioSpin GmbH, Rheinstetten, Germany). All juice and peel extracts were analyzed at 300.1 ± 0.1 K without sample rotation. Four test scans were performed prior to acquisition, followed by 32 scans per sample. Acquisition parameters were as follows: FID size of 64 K, spectral width of 12.4345 ppm, receiver gain of 28.5, acquisition time of 2.18 s, relaxation delay of 2 s, and line broadening of 0.50 Hz. Spectra were acquired using the NOESY pulse sequence with presaturation (Bruker 1D, noesypr1d), applying water signal suppression by irradiation at the water resonance frequency during the recycling and mixing periods.

Each spectrum was individually processed using noise reduction based on multilevel signal deconvolution, followed by baseline correction and signal area interpolation. This procedure generated a representative spectral fingerprint for each sample, providing an overview of the most abundant metabolites present at harvest, expressed as chemical shifts (δ) in parts per million (ppm). All spectra were calibrated using the internal standard signal of trimethylsilylpropionic acid-d4 (TSP-d4), and the pH was adjusted to a value close to 6.

The resulting ^1^H-NMR spectra were processed using Chenomx NMR Suite software version 8.3 (Chenomx, Edmonton, AB, Canada) for metabolite identification and relative quantification. Data processing included baseline correction, spectral alignment, and peak determination [34,35]. Identification was based on comparison with reference spectra of pure metabolites. Metabolite quantification was performed on individual, non-overlapping peaks whenever possible, and peaks were characterized according to their chemical shift (ppm), multiplicity, and coupling constants (Hz).

The software includes an extensive spectral database that enables the detection of a wide range of metabolites. Only metabolites quantified above the established detection (5–10 μM) and quantification (>3 μM) limits for this study were included in the analysis, ensuring the robustness and reproducibility of the comparative metabolomic profiling.

### 2.6. Data Analysis and Statistics

Metabolite identification and relative quantification were performed using Chenomx NMR Suite v11 software (Chenomx Inc., Edmonton, AB, Canada). For statistical analyses, data are presented as means ± standard deviation. Univariate analyses were conducted using one-way analysis of variance (ANOVA) and Student’s *t*-test for pairwise comparisons, implemented in R software v4.2.2 (R Foundation for Statistical Computing, Vienna, Austria).

Regarding the physical parameter measurements of trees across treatments, treatment *T5* differed from the others in both sample size (*n* = 28 vs. *n* = 21) and in the timing of weed control netting implementation. Therefore, all analyses involving this treatment were conducted using statistical approaches appropriate for unequal sample sizes and potential heteroscedasticity. Specifically, when comparing all six treatments (*T0*–*T5*), data were analyzed using Welch’s heteroscedastic one-way ANOVA followed by Games–Howell post hoc tests, which do not assume equal variances or balanced designs. In contrast, confirmatory comparisons presented in the tables correspond to the balanced experimental set (*T0*–*T4*) and were analyzed using a standard one-way ANOVA followed by Tukey’s HSD test, as originally planned for homogeneous groups. This approach ensures transparency, methodological consistency, and reproducibility of the reported results.

Multivariate analyses were performed using MetaboAnalyst 6.0 (Xia Lab, McGill University, Montreal, QC, Canada), including principal component analysis (PCA), partial least squares discriminant analysis (PLS-DA), and significance analysis of microarrays (SAM), to explore metabolic differences among treatments. Default platform parameters were applied, including mean centering and Pareto scaling of variables, and two principal components were selected for visualization. In PLS-DA models, variable relevance was assessed using variable importance in projection (VIP) scores, with metabolites showing VIP > 1 considered influential contributors to group separation, and model performance was evaluated using R^2^ and Q^2^ values.

Given the limited number of biological replicates, multivariate analyses were used in an exploratory manner to support data visualization and pattern recognition, and biological interpretation was primarily based on consistent trends supported by univariate statistical analyses.

## 3. Results and Discussion

### 3.1. Initial and Final Evolution of the Vegetative State of the Trees

The mean height, crown diameter, and trunk diameter values are shown in Table 2 for the two assessment dates (19 June 2024 and 17 December 2024).

In both samplings, no significant differences in height were detected between treatments (same letters, *p* < 0.05), with ranges from 108.00 ± 20.57 cm (*T1*) to 121.57 ± 14.83 cm (*T3*) in June, and from 137.67 ± 18.71 cm (*T0*) to 152.28 ± 20.56 cm (*T4*) in December. This suggests that height, at this growth stage, is less sensitive to the management practices evaluated.

However, crown and trunk diameter did respond to the treatments. In June, all net treatments (*T1*–*T5*) showed significantly larger crown diameters than the *T0* control (e.g., *T4*: 112.40 ± 14.01 cm vs. *T0*: 92.31 ± 12.07 cm; ≈+22%). Similarly, trunk diameter was larger in *T4* (28.57 ± 4.08 mm) than in *T0* (22.27 ± 2.57 mm; ≈+28%). In December, the pattern continued: *T4* reached the largest canopy (166.77 ± 16.83 cm), significantly larger than *T0* (150.95 ± 12.98 cm; ≈+11%), and had the largest trunk (40.19 ± 4.40 mm vs. 36.37 ± 4.93 mm in *T0*; ≈+10%), while the remaining treatments did not differ in trunk size.

Overall, the results support the positive effect of the weed control mesh on structural growth (canopy and, to a lesser extent, trunk), probably due to a reduction in soil water loss, an improvement in the root microclimate, and greater water use efficiency. *T4* (mesh + zeolite) stands out as the most consistent management, which is consistent with the literature, where it is attributed to zeolite improvements in water retention and nutrient availability in calcareous/saline soils, favoring vegetative development [7,36,37,38,39].

### 3.2. Initial Fruit Production, Thinning, and Final Fruit Production

Table 3 presents the mean values for the initial number of fruits, thinning performed, final number of fruits harvested, and total yield by treatment. Statistically significant differences (*p* < 0.05) were observed in all variables between the traditional treatment (*T0*) and the netting treatments (*T1*–*T4*).

The *T0* control showed the lowest performance, with 8.43 ± 5.83 initial fruits, minimal thinning (1.81 ± 3.43), and only 5.95 ± 3.07 fruits harvested per tree, which translated into the lowest total yield (46.69 kg). This confirms that traditional management is less efficient at promoting fruit set and yield under the soil and climate conditions of the trial. In contrast, net treatments *T1*, *T2*, *T3*, and *T4* had initial fruit loads 3–4 times higher than *T0*, requiring considerable thinning to homogenize fruit load between treatments.

*T1* recorded the highest total production (69.90 kg, ≈+50% compared to *T0*), with 9.71 ± 2.15 final fruits per tree. *T2* and *T3* performed similarly, with 29.90 ± 8.97 and 29.67 ± 9.58 initial fruits, respectively, and total yields of 65.13–65.76 kg (≈+40% compared to *T0*). *T4* stood out for its highest initial crop load (34.19 ± 20.26 fruits), which required the most thinning (24.48 ± 19.85), while still maintaining 9.33 ± 1.80 final fruits and a yield of 61.33 kg. This performance indicates that the combination of netting and zeolite enhances fruit set, although thinning must be carefully managed to avoid excessive competition for resources. *T5* had similar results to *T0* in terms of the number of fruits and fruits harvested, although with slightly higher yield (57.71 kg), suggesting that netting without drainage or amendments offers a partial benefit.

Overall, treatments *T1*–*T4* significantly increased productivity per tree, confirming the usefulness of weed control netting and its combination with drainage systems and soil amendments as management tools to optimize yield. These findings are consistent with previous studies documenting that improved water conditions and reduced competition with weeds favor fruit set, fruit growth, and total yield [14,16,40].

### 3.3. Physical Parameters of the Fruit

Analysis of the physical characteristics of the fruits (Table 4) revealed no significant differences in external morphology between treatments.

All fruits were seedless, with an average of 10.88 segments per fruit, a value consistent with that reported in previous studies [15,41,42]. The average skin thickness was 4.29 mm, with no significant differences between treatments, in line with data described by Domínguez-Gento et al. and Ortiz [43,44].

Regarding fruit size and weight, notable differences were found. Fruits from the traditional treatment (*T0*) had the highest average weight (385.18 ± 59.02 g), exceeding those obtained from the weed-netting treatments (*T1*–*T5*) by approximately 19%. This greater weight was associated with larger equatorial and polar diameters (88.59 ± 4.57 mm and 88.47 ± 4.70 mm, respectively), suggesting a more spherical morphology. This type of conformation can be advantageous for commercial packaging and industrial processing by facilitating more uniform sizing and more efficient fruit handling [15,45,46].

Fruits from *T0* also had the heaviest rind and the highest absolute juice volume. However, when analyzing the percentage of juice yield, the weed-netting treatments showed slightly higher values, with percentages ranging from 53.64% (*T1*) to 55.84% (*T2*), compared to 51.34% recorded in *T0*. This indicates that, although the fruits from the traditional system are larger and heavier, they have a higher proportion of rind, which reduces the usable juice fraction. In contrast, the netting treatments, especially *T4* (zeolite-infused netting) and *T2*, showed the highest weight-to-rind ratios (2.68 and 2.46, respectively), suggesting a greater allocation of metabolites to endocarp development and, consequently, greater efficiency in juice production per unit of fresh biomass.

This finding is particularly relevant to the agri-food industry, as it suggests that implementing weed-repellent nets can optimize juice extraction yield, reduce the volume of by-products generated, and thus improve overall process efficiency. From a production perspective, a trade-off is observed between fruit size and juice yield: the traditional system favors larger fruits, suitable for the fresh fruit market, while netting treatments produce smaller fruits but with a higher percentage of juice, a desirable characteristic for industrial processing. The choice of handling system could therefore be tailored to the final destination of the produce, optimizing the commercial value of the harvest.

### 3.4. Metabolomic Profile of the Peel

A total of 23 major metabolites were detected in the ^1^H-NMR profiling of the peel (Table 5). Although additional signals were observed at very low levels, these compounds could not be reliably quantified with the software used because they were below the established detection limits, which is consistent with what was described by Xie et al. [47]. Of the 23 metabolites identified, nine corresponded to amino acids, eight to organic acids, four to sugars, and two to other functional groups. Overall, sugars accounted for the predominant biochemical fraction in the peel, representing approximately 84.05% of the total quantified metabolites, followed by organic acids (10.22%), amino acids (4.76%), and other metabolites (0.95%).

Mean concentrations indicated that glucose and fructose were the predominant sugars, malate and quinic acid were the most abundant organic acids, and asparagine was the most abundant amino acid. In total, 16 of the 23 quantified metabolites showed significant differences among treatments, including five amino acids (asparagine, glutamate, phenylalanine, proline, and leucine), five organic acids (ascorbate, citrate, lactate, malate, and quinic acid), four sugars (fructose, glucose, myo-inositol, and sucrose), and two metabolites from other groups (choline and ethanol). These results indicate that soil management practices based on weed-control netting and associated amendments can modulate the peel metabolome, affecting sugars, amino acids, and antioxidant-related compounds [48].

#### 3.4.1. Principal Component Analysis:Differentiation of Orange Peel Samples

The results in this section were obtained using the MetaboAnalyst 6.0 platform, which is widely used for metabolomic data analysis and multivariate visualization. In this study, one-way comparisons were applied to evaluate peel and juice samples across treatments (*T0*, *T1*, *T2*, *T3*, *T4*, and *T5*), and paired approaches were used when appropriate, supporting an integrated assessment of metabolomic variation among treatments and within each group.

Principal component analysis (PCA) was applied as an unsupervised chemometric method to explore the overall structure of the dataset and to evaluate sample grouping patterns and potential outliers. PCA reduces data dimensionality by transforming the original variables into a limited number of principal components that retain most of the variance [49], thereby facilitating visualization and interpretation [50].

**Figure 1:**
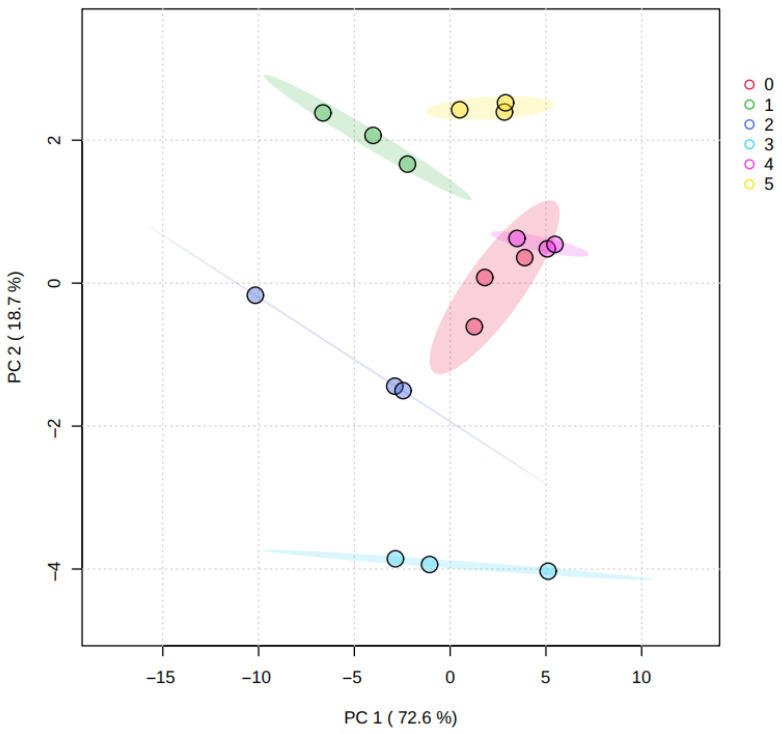
Principal component analysis graph, representing the metabolomic profiles of the fruit peel from all the treatments (0. ‘*T0*’; 1. ‘*T1*’; 2. ‘*T2*’; 3. ‘*T3*’; 4. ‘*T4*’; 5. ‘*T5*’). Colored shaded areas represent confidence ellipses for each treatment group.

Figure 1 shows the PCA results for peel samples, where Principal Component 1 (PC1) explained 72.6% of the total variability, and Principal Component 2 (PC2) explained 18.7%; together, these components accounted for 91.3% of the variance. This proportion indicates that the first two components capture most of the information contained in the dataset. Replicates from the same treatment clustered consistently, and no samples were identified as outliers within the confidence regions shown in the plot.

Along the PC2 axis, samples from treatments *T3* and *T5* exhibited the largest separation, whereas most samples were closer along the PC1 axis. This distribution suggests that treatment-related differences are mainly associated with a subset of metabolites contributing to PC2, while the overall peel metabolic profile remains broadly conserved across treatments.

**Figure 2:**
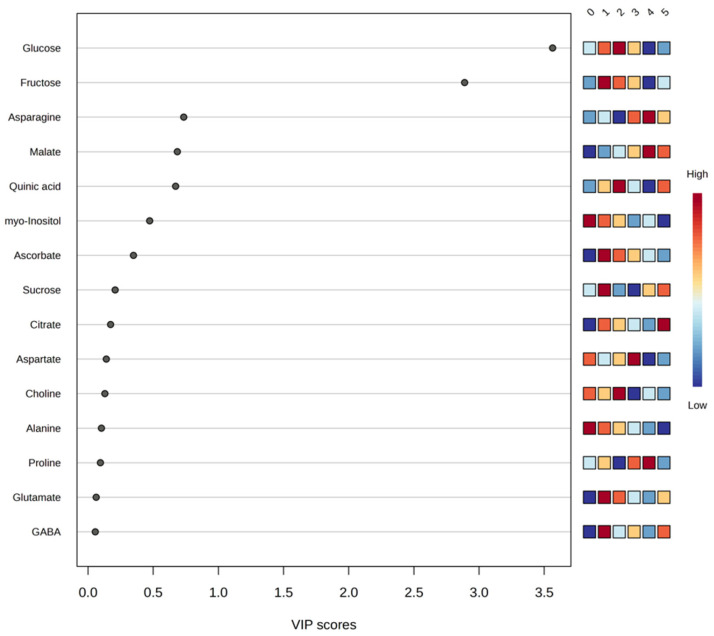
Graphical representation of partial least squares discriminant analysis (PLSD-DA) using a variable importance projection (VIP) plot of the set of most significant metabolites detected in orange peel across all study theses of a factor. The colored boxes on the right indicate the relative concentrations of the corresponding metabolite in each study treatment, with red being the color representing the highest concentration of each metabolite (0. ‘*T0*’; 1. ‘*T1*’; 2. ‘*T2*’; 3. ‘*T3*’; 4. ‘*T4*’; 5. ‘*T5*’).

PLS-DA was used as a supervised multivariate approach to facilitate visualization of treatment separation based on metabolomic signatures and to identify metabolites contributing to class discrimination through VIP scoring. The results indicated that glucose, fructose, asparagine, malate, and quinic acid, which were among the most abundant peel metabolites, contributed notably to the multivariate separation pattern and displayed VIP values above 0.5 (Figure 2), supporting their relevance to the observed differentiation.

The PLS-DA model was evaluated using accuracy, R^2^ (explained variance), and Q^2^ (predictive ability). For the three-component model, cumulative values were as follows: Accuracy = 0.59333, R^2^ = 0.62928, and Q^2^ = 0.34454. To assess whether the observed discrimination exceeded what could be expected by chance, a permutation test with 2000 permutations was performed (*p* < 5 × 10^−4^). Consistent with the statistical analysis framework described above, these supervised multivariate results are interpreted as exploratory and are used to support visualization and variable prioritization rather than predictive inference.

Overall, the multivariate patterns suggest that treatments combining weed-control netting with soil amendments, such as zeolite, are associated with shifts in peel metabolites linked to primary metabolism and stress-related responses, which may influence the nutritional and functional traits of the fruit.

#### 3.4.2. Amino Acids

Among amino acids showing significant differences, asparagine presented the highest concentrations in treatments *T3* (2.21 mM) and *T4* (2.23 mM), values above the overall mean across treatments. This increase may be related to the higher vegetative development observed under these treatments, which recorded higher values for height, crown diameter, and trunk diameter during the experimental period. Asparagine plays a central role in nitrogen transport and storage, and its synthesis is enhanced in tissues with high nitrogen demand for growth. Previous studies indicate that its biosynthesis is regulated by light in leaves [51,52,53]. In this context, it is relevant that orange peel remains photosynthetically active during much of fruit development, which may contribute to asparagine accumulation under these management conditions.

For glutamate, samples showed a separation pattern associated with the presence of weed-control netting. The lowest concentration was detected in *T0* (0.40 mM), which was 0.29 mM below the mean of netted treatments. This observation suggests that weed-control netting may favor glutamate synthesis or accumulation in peel, potentially through improved root microclimate and nitrogen availability [8,9,10,54]. This interpretation is consistent with the greater vegetative growth observed in *T3* and *T4* and with the role of glutamate as a central metabolite in plant nitrogen metabolism and a precursor of other amino acids [55,56,57].

Phenylalanine and leucine were detected at low concentrations across treatments. Although differences were statistically significant, their low variability and the absence of consistent associations with agronomic parameters limited biological interpretation in the present dataset.

Proline was the second most abundant amino acid in peel and reached the highest values in *T3* (1.36 mM) and *T4* (1.65 mM). This finding aligns with the established role of proline in stress response, osmotic regulation, cellular protection, and antioxidant defense in plants [58,59,60,61]. Higher proline levels in these treatments may reflect differences in tissue growth dynamics and osmotic regulation requirements.

#### 3.4.3. Organic Acids

Within organic acids, quinic acid was detected at the highest concentrations across treatments, with *T2* showing the highest value (8.66 mM) and *T4* the lowest (5.46 mM). The lower concentration in *T4* may be associated with the incorporation of zeolites, which can modify nutrient and water dynamics in soil and thereby influence plant metabolism [7,62]. Quinic acid has also been reported as one of the most abundant metabolites in foliar metabolomics studies of sweet orange [10]. Given the protective role of peel tissues, which remain photosynthetically active from fruit set to ripening, quinic acid may be linked to defense-related functions and the synthesis of protective compounds against biotic agents [63,64,65,66].

Formate, fumarate, and succinate remained stable across treatments and did not display patterns that supported differentiation. In contrast, ascorbate, citrate, and malate tended to show higher concentrations in treatments with weed-control netting, suggesting that this management practice may favor their accumulation in peel. These metabolites participate in core metabolic processes, including respiration, the tricarboxylic acid cycle, and antioxidant responses [12,67,68,69]. Lactate showed the opposite pattern, with the highest concentration in *T0*. The absence of netting could have promoted episodes of water imbalance or osmotic stress, potentially increasing lactate levels, a metabolite involved in intermediary metabolism and stress-related regulation of cellular pH [70,71].

#### 3.4.4. Sugars

Sugars constituted the most abundant fraction of the peel’s metabolomic profile. Fructose was the predominant sugar across treatments, followed by glucose, sucrose, and myo-inositol. The highest fructose concentration was detected in *T2* (42.00 mM), whereas *T4* showed the lowest value (35.15 mM). Except for *T4*, treatments with weed-control netting presented higher fructose concentrations than the traditional treatment, suggesting that netting may favor fructose accumulation under the conditions tested.

A comparable trend was observed for glucose, although differences were less marked; again, *T4* recorded the lowest concentration, indicating that the presence of zeolite may modulate sugar synthesis or accumulation in peel. For myo-inositol and sucrose, no consistent treatment-related patterns were observed that supported robust conclusions within the present dataset.

#### 3.4.5. Other Metabolites

Within the group of other metabolites, including choline and ethanol, no consistent differences were identified that could be associated with a specific treatment. Concentrations remained within similar ranges across treatments, suggesting limited sensitivity of these compounds to the management systems evaluated under the present conditions.

### 3.5. Metabolomic Profile of the Juice

Metabolomic analysis of juice identified 21 major metabolites (Table 6). Although additional signals were detected, their concentrations were very low and not reliably quantifiable with the software used; therefore, they were not included in the statistical analysis. Of the 21 quantified metabolites, 11 corresponded to amino acids [72,73], five to organic acids, four to sugars, and one to other metabolites. In relative terms, sugars represented the predominant fraction of the juice metabolome (approximately 79.65% of the total), followed by organic acids (14.29%), amino acids (5.99%), and other metabolites (0.05%).

Among quantified metabolites, 16 showed significant differences among treatments, including eight amino acids (alanine, asparagine, aspartate, arginine, glutamate, glutamine, isoleucine, and proline), three organic acids (ascorbate, citrate, and succinate), four sugars (fructose, glucose, myo-inositol, and sucrose), and choline as the only metabolite in the “Others” group. Comparison of mean concentrations indicated that sucrose, glucose, and fructose were predominant in juice, while citrate was the most abundant organic acid. Among amino acids, asparagine and arginine were detected at notable concentrations, consistent with their role in nitrogen transport and in growth and stress-related regulation [48].

#### 3.5.1. Principal Component Analysis: Differentiation of Orange Juice Samples

**Figure 3:**
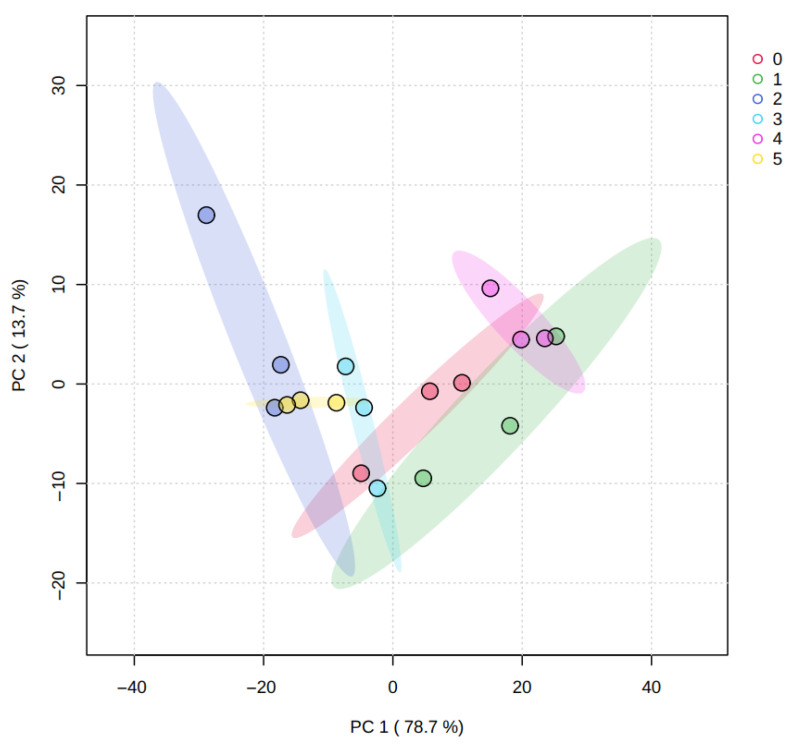
Principal component analysis graph, representing the metabolomic profiles of the orange juice from all the treatments (0. ‘*T0*’; 1. ‘*T1*’; 2. ‘*T2*’; 3. ‘*T3*’; 4. ‘*T4*’; 5. ‘*T5*’). Colored shaded areas represent confidence ellipses for each treatment group.

Figure 3 shows the PCA results for juice metabolomic profiles. PC1 explained 78.7% of the total variability, while PC2 accounted for 13.7%, with both components together explaining 92.4% of the variance. This indicates that the first two components capture most of the dataset structure. No samples fell outside the confidence intervals, supporting internal consistency and the absence of outliers.

Juice samples tended to form tight clusters by treatment, with less dispersion and partial overlap among groups compared to peel, suggesting that treatment-related metabolomic differences were generally less pronounced in juice. Nevertheless, treatments *T2* and *T1* were positioned further apart along the principal components, indicating comparatively greater differentiation in their juice metabolic composition under the tested management conditions.

**Figure 4:**
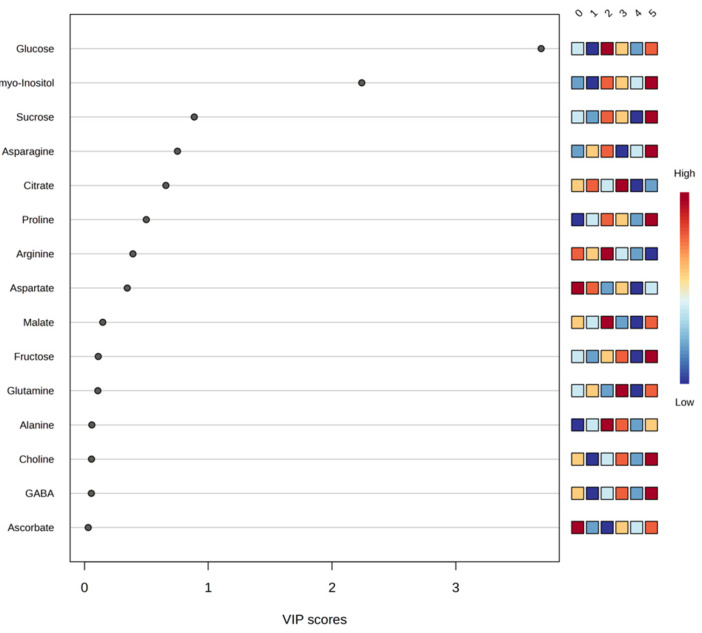
Graphical representation of partial least squares discriminant analysis (PLSD-DA) using a variable importance projection (VIP) plot of the set of most significant metabolites detected in orange juice across all study theses of a factor. The colored boxes on the right indicate the relative concentrations of the corresponding metabolite in each study treatment, with red being the color representing the highest concentration of each metabolite (0. ‘*T0*’; 1. ‘*T1*’; 2. ‘*T2*’; 3. ‘*T3*’; 4. ‘*T4*’; 5. ‘*T5*’).

PLS-DA was used to facilitate supervised visualization of group separation and to prioritize discriminant variables based on VIP scoring. Metabolites contributing notably to the observed differentiation included glucose, myo-inositol, sucrose, asparagine, citrate, proline, arginine, and aspartate, which displayed VIP values above 0.5 (Figure 4). These metabolites suggest that differences in sugar and nitrogen metabolism and in tricarboxylic-acid-cycle-related compounds contribute to the observed multivariate separation pattern.

Model evaluation yielded cumulative performance values for the four-component model of Accuracy = 0.53333, R^2^ = 0.7429, and Q^2^ = 0.24188, indicating limited predictive ability. A permutation test with 2000 permutations resulted in *p* = 0.0225, suggesting that class discrimination exceeded random expectation. However, despite statistical significance, the low Q^2^ supports interpreting the model as exploratory, useful for visualization and variable prioritization rather than for prediction.

Overall, these results indicate that soil management and weed-control netting are associated with shifts in juice metabolite composition, affecting sugars, amino acids, and organic acids. These variations may influence industrially relevant quality traits, including total sugars, acidity profile, and organoleptic balance, emphasizing the relevance of soil management to citrus chemical quality.

#### 3.5.2. Amino Acids

Of the eleven amino acids detected in the juice, eight showed statistically significant differences between treatments (alanine, asparagine, aspartate, arginine, glutamate, glutamine, isoleucine, and proline), while GABA, leucine, and valine did not show significant variations.

Asparagine was the most abundant amino acid, with notable variability between theses: *T5* had the highest concentration (9.54 mM), in contrast to *T3*, which recorded the lowest (3.42 mM). This marked difference suggests that the gravel trench cultivation system used in *T3* may limit nitrogen availability in the root profile, reducing asparagine accumulation in the fruit [74,75]. This result is consistent with studies indicating that soluble asparagine accumulates preferentially when there is excess nitrogen not destined for protein synthesis [51,52,53,76].

Alanine, glutamate, glutamine, and proline concentrations showed patterns that clearly differentiated treatments with weed control netting from those without, suggesting that this management practice influences the synthesis and accumulation of amino acids related to nitrogen metabolism and stress response. It is noteworthy that *T4*, despite being covered with netting, presented a differentiated profile due to the application of zeolite, confirming that this amendment specifically modulates nitrogen and carbon metabolism in the fruit [36,37,39]. Alanine, in particular, is an amino acid precursor to other nitrogenous molecules, and its accumulation could be related to the greater vegetative development observed in the netted plants, which showed higher values for height, crown diameter, and trunk compared to the traditional treatment [77,78,79].

Regarding aspartate, arginine, and isoleucine, although significant differences were detected between treatments, no clear correlation could be established with the physiological or growth parameters evaluated, so their interpretation should be considered with caution and may require additional studies to confirm their biological relevance in the context of the management conditions tested.

#### 3.5.3. Organic Acids

As expected in orange juice, citrate was the predominant organic acid in all treatments, reaching its highest concentration in *T3* (32.01 mM) and lowest in *T4* (23.77 mM) [80]. Citrate was up to three times more abundant than malate, which ranked second in concentration among the organic metabolites. The difference of approximately 5 mM between *T3* and the other treatments suggests that the gravel trench used in *T3* could significantly promote citrate synthesis or accumulation in the fruit [81]. In contrast, the application of zeolites in *T4* appeared to reduce its concentration, which is consistent with the modulating effect that these amendments have on cation availability and soil pH [36,37,39].

Malate behavior was similar to that observed for citrate, with *T4* recording the lowest concentration (9.20 mM), which could be associated with a lower accumulation of tricarboxylic acids under this treatment [68]. However, succinate showed the opposite pattern, reaching its maximum concentration in *T4* (0.08 mM), which could indicate a shift in the metabolic balance of Krebs cycle intermediates under this treatment [66,82].

In the case of lactate, no statistically significant differences were detected between treatments, suggesting that this metabolite remains relatively stable regardless of management practices. Ascorbate, on the other hand, showed significant variations between treatments, but the high dispersion of values made it difficult to establish solid correlations with other physiological or fruit quality parameters.

#### 3.5.4. Sugars

The sugar group was, as in the peel, the most quantitatively representative in the juice. Sucrose was the majority sugar in all treatments, followed by fructose, glucose, and myo-inositol, with little marked difference between fructose and glucose, indicating a relatively uniform balance between the two carbohydrates.

Fructose levels varied significantly between treatments. *T5* had the highest concentration (74.02 mM), followed by *T3* (73.66 mM), while *T4* had the lowest (58.86 mM) [83,84,85]. This result suggests that the addition of zeolite to *T4* could have modulated the accumulation of simple sugars in the fruit, affecting its metabolic profile [7,36,86]. Regarding glucose, *T2* showed the highest concentration (86.90 mM), making it the most effective treatment for maintaining high levels of this metabolite, followed by *T5* (75.52 mM) and *T3* (68.42 mM), while *T1* and *T4* had the lowest values (51.23 mM and 52.88 mM, respectively).

Myo-inositol also showed notable differences between treatments. *T5* recorded the highest concentration (7.64 mM), followed by *T2* (5.97 mM) and *T3* (5.96 mM), while *T1* had the lowest (1.26 mM), which could be related to management conditions and the availability of precursors for its synthesis [84,87,88].

In the case of sucrose, *T5* had the highest concentration (92.79 mM), followed by *T2* (91.54 mM) and *T3* (87.68 mM), while *T4* recorded the lowest (73.87 mM). The high concentration of sugars in *T5* could be explained by the particular conditions of this experimental block: at the beginning of fruit set, the net treatment produced the fewest fruits, required the least thinning, and consequently maintained a lower yield load, which probably allowed for greater carbohydrate accumulation per fruit. Furthermore, *T5* was harvested slightly later than the other treatments, a factor that may have also favored ripening and final sugar accumulation.

In contrast, the incorporation of zeolite in *T4* appears to have reduced carbohydrate synthesis or accumulation, consistent with studies reporting that zeolites, by acting as nutrient reservoirs, can modify the availability of potassium and other cations essential for sugar translocation and metabolism [7,62]. Their ability to improve soil water retention may also have reduced osmotic stress in this treatment, modifying hormonal balance and the flow of assimilates to the fruit.

#### 3.5.5. Other Metabolites

In orange juice, choline was the only metabolite classified within the “other” group that was detected in quantifiable concentrations. Treatment *T5* had the highest concentration (0.31 mM), significantly higher than the other treatments, suggesting that the specific conditions of this cultivation system, characterized by the use of mesh, a lower number of fruits from the start, and a larger canopy volume per fruit, favored the synthesis or accumulation of choline in the fruit.

Treatment *T3* showed an intermediate value (0.18 mM), although without significant differences compared to *T0*, *T1*, *T2*, and *T4*. Choline is an essential metabolite in plant metabolism, key in the biosynthesis of phosphatidylcholine for membrane formation and in cell signaling pathways [87]. Its accumulation can be modulated by the availability of nutrients, the presence of abiotic stress, and the physiological conditions of the plant [88], so the result observed in *T5* could be associated with a favorable combination of these factors.

### 3.6. Comparative Study of Skin/Juice

The comparative analysis of peel and juice fractions (Table 7) revealed consistent patterns in the distribution of major biochemical groups. For amino acids, total concentrations in peel ranged from 4.92 to 6.34 mM, whereas higher values were observed in juice (15.56 to 20.62 mM), indicating that amino acids were, on average, 3.13-fold more concentrated in juice than in peel. This distribution is consistent with a higher representation of soluble nitrogen compounds in the liquid fraction.

A similar trend was observed for organic acids: peel values ranged from 10.55 to 15.15 mM, while juice concentrations ranged from 33.41 to 43.32 mM, approximately threefold higher. This enrichment aligns with the role of organic acids in defining citrus juice acidity and characteristic flavor.

Sugars were the predominant group in both fractions, but were markedly higher in juice. Peel sugar totals ranged from 83.19 to 97.86 mM, whereas juice totals ranged from 190.92 to 257.45 mM, corresponding to an average 2.52-fold increase. This is consistent with juice being the main reservoir of soluble carbohydrates, contributing directly to nutritional and sensory attributes.

Other metabolites were present at very low concentrations in both fractions (below 1 mM in all cases), indicating a marginal contribution to the total metabolite pools. Collectively, these results confirm that juice contains higher concentrations of amino acids, organic acids, and sugars than peel [88]. The juice/peel ratios (2.36–2.88 for total metabolites) further highlight juice as the main compartment for the accumulation of metabolites relevant to organoleptic quality and nutritional value in citrus production.

## 4. Conclusions

The results of this study indicate that weed-control netting, particularly when combined with zeolite-amended soil (*T4*), is associated with consistent improvements in vegetative growth and fruit-related parameters compared with the traditional management system (*T0*). Although tree height did not differ significantly between treatments at either assessment date, canopy development and, to a lesser extent, trunk diameter tended to be higher under netting-based systems, with *T4* showing the most consistent responses across sampling dates. These findings support the agronomic relevance of weed-control netting and suggest that zeolite may contribute to improved water and nutrient dynamics under the soil conditions of the trial.

In terms of fruit production and physical traits, the traditional system (*T0*) produced heavier and larger fruits, which may be advantageous for packing and certain processing operations, but it showed a lower initial fruit load and overall yield. In contrast, netting-based systems improved juice production efficiency by increasing juice yield percentage and reducing the relative proportion of peel, thereby potentially decreasing by-product generation per unit of juice. Accordingly, netting treatments (*T1*–*T5*) appear more suitable when the production goal prioritizes juice yield and resource-use efficiency, whereas *T0* may be more aligned with strategies favoring larger fruit size.

Comparative ^1^H-NMR metabolomics profiling further showed that juice contained markedly higher concentrations of amino acids, organic acids, and sugars than peel, highlighting the juice fraction as the main reservoir of soluble metabolites relevant to organoleptic and nutritional attributes. Treatment-related differences in the metabolomic dataset were interpreted as comparative trends, supported primarily by univariate statistics, with multivariate analyses used to assist visualization and pattern recognition. Overall, the combined agronomic and metabolomic evidence suggests that weed-control netting, particularly when integrated with zeolite, can modulate tree performance and fruit composition in ways that may benefit productivity and processing-oriented quality targets. These findings provide a practical basis for considering such management strategies in citrus production programs aimed at profitability, sustainability, and improved utilization of agro-industrial by-products.

## Figures and Tables

**Table 1:** Description of the culture conditions that were applied in the different experimental thesis/study blocks.

Treatment	Anti-Weed Mesh	Underground Drainage	Others	Observations
*T0*	No	No	No	Traditional farming system Control-control thesis
*T1*	Yes	Yes, located between the rows of trees	No	Simulation of consolidated plantations with adult trees
*T2*	Yes	Yes, located below the rows of trees	No	Simulation of new plantations
*T3*	Yes	Yes, located below the rows of trees	Gravel ditch	Simulates the traditional drainage system
*T4*	Yes	Yes, located below the rows of trees	Zeolite	Add natural soil improvers
*T5*	Yes	No	No	Traditional mulching cultivation system

**Table 2:** Average values of the physical parameters height (cm), crown diameter (cm), and trunk diameter (mm), measured on all the trees that make up the study in June and December 2024. % Δ *T0* Cd: percentage increase in crown diameter growth relative to *T0*. % Δ *T0* Td: percentage increase in trunk diameter growth relative to *T0*.

Treatment	Date	Height (cm)	Crown Diameter (cm)	Trunk Diameter (mm)	% Δ *T0* Cd	% Δ *T0* Td
*T0*	19 June 2024	112.52 ± 15.76 ^a^	92.31 ± 12.07 ^a^	22.27 ± 2.57 ^a^	0.00	0.00
*T1*	19 June 2024	108.00 ± 20.57 ^a^	106.86 ± 10.90 ^b^	24.93 ± 4.74 ^a,b^	15.76	11.94
*T2*	19 June 2024	116.14 ± 13.74 ^a^	110.38 ± 12.42 ^b^	25.23 ± 3.87 ^a,c^	19.58	13.29
*T3*	19 June 2024	121.57 ± 14.83 ^a^	107.88 ± 12.88 ^b^	26.43 ± 4.02 ^b,c^	16.87	18.68
*T4*	19 June 2024	118.67 ± 15.64 ^a^	112.40 ± 14.01 ^b^	28.57 ± 4.08 ^c^	21.76	28.29
*T5*	19 June 2024	113.64 ± 18.68 ^a^	106.33 ± 11.88 ^b^	24.54 ± 3.52 ^a,b^	15.19	10.19
*T0*	17 December 2024	137.67 ± 18.71 ^a^	150.95 ± 12.98 ^a^	36.37 ± 4.93 ^a^	0.00	0.00
*T1*	17 December 2024	142.48 ± 16.41 ^a^	156.93 ± 15.30 ^a,b^	36.88 ± 5.86 ^a^	3.96	1.40
*T2*	17 December 2024	139.33 ± 17.49 ^a^	152.92 ± 18.56 ^a,b^	36.28 ± 7.17 ^a^	1.31	−0.25
*T3*	17 December 2024	152.28 ± 19.18 ^a^	156.75 ± 20.02 ^a,b^	37.38 ± 5.86 ^a^	3.84	2.78
*T4*	17 December 2024	152.28 ± 20.56 ^a^	166.77 ± 16.83 ^b^	40.19 ± 4.40 ^a^	10.48	10.50
*T5*	17 December 2024	140.68 ± 19.82 ^a^	148.21 ± 16.01 ^a^	35.63 ± 5.43 ^a^	−1.82	−2.03

Note: Values are expressed as mean ± standard deviation. Different lowercase letters within the same column indicate significant differences among treatments at *P* < 0.05 (one-way ANOVA followed by Tukey’s HSD test). The number of samples was *n* = 21 for all treatments (*T0*–*T4*), except *T5*, for which *n* = 28.

**Table 3:** Average values of the initial number of fruits produced (Units), the average thinning carried out (Units), the average of the fruits finally harvested (Units), and the total production (kg) and its average weight (g) of the 2024 campaign.

Treatment	N° of Fruits (Units)	Thinning (Ud.)	Harvested Fruits (Units)	Total Production (kg)
*T0*	8.43 ± 5.83 ^a^	1.81 ± 3.43 ^a^	5.95 ± 3.07 ^a^	46.69
*T1*	26.57 ± 12.29 ^b^	16.95 ± 11.75 ^b^	9.71 ± 2.15 ^b^	69.90
*T2*	29.90 ± 8.97 ^b^	19.90 ± 8.97 ^b^	10.43 ± 2.56 ^b^	65.13
*T3*	29.67 ± 9.58 ^b^	19.67 ± 9.58 ^b^	10.09 ± 1.26 ^b^	65.76
*T4*	34.19 ± 20.26 ^b^	24.48 ± 19.85 ^b^	9.33 ± 1.80 ^b^	61.33
*T5*	9.25 ± 6.23 ^a^	2.14 ± 3.86 ^a^	6.25 ± 2.94 ^a^	57.71

Note: Values are expressed as mean ± standard deviation. Different lowercase letters within the same column indicate significant differences among treatments at *p* < 0.05 (one-way ANOVA followed by Tukey’s HSD test). The number of samples was *n* = 21 for all treatments (*T0*–*T4*), except *T5*, for which *n* = 28.

**Table 4:** Average physical parameters recorded on the fruits produced by the different treatments in the 2024 campaign.

Treatment	Fruit Weight (g)	Equatorial Diameter (mm)	Polar Diameter (mm)	Skin Thickness (mm)	Juice Volume (mL)	Peel Weight (g)	Juice Yield (%)
*T0*	385.18 ± 59.02 ^b^	88.59 ± 4.57 ^b^	88.47 ± 4.70 ^a^	4.37 ± 1.00 ^a^	197.60 ± 30.67 ^a^	158.97 ± 30.86 ^b^	51.34 ± 3.58 ^a^
*T1*	333.86 ± 37.21 ^a^	86.31 ± 3.70 ^a,b^	85.31 ± 3.74 ^a^	4.369 ± 0.61 ^a^	178.33 ± 17.83 ^a^	136.27 ± 17.35 ^a^	53.64 ± 4.30 ^a^
*T2*	311.73 ± 39.14 ^a^	83.22 ± 3.61 ^a^	84.94 ± 3.89 ^a^	3.97 ± 0.66 ^a^	173.87 ± 25.06 ^a^	126.15 ± 17.98 ^a^	55.84 ± 4.84 ^a^
*T3*	331.25 ± 40.46 ^a^	85.03 ± 3.71 ^a,b^	88.08 ± 6.62 ^a^	4.28 ± 0.86 ^a^	179.33 ± 21.44 ^a^	131.80 ± 18.66 ^a^	54.26 ± 3.64 ^a^
*T4*	317.05 ± 45.47 ^a^	83.03 ± 3.89 ^a^	87.11 ± 3.99 ^a^	4.17 ± 0.83 ^a^	176.13 ± 34.32 ^a^	118.74 ± 15.84 ^a^	55.63 ± 8.85 ^a^
*T5*	323.25 ± 48.70 ^a^	84.74 ± 4.07 ^a,b^	86.35 ± 4.38 ^a^	4.59 ± 0.95 ^a^	175.60 ± 36.49 ^a^	123.67 ± 17.44 ^a^	54.31 ± 7.82 ^a^

Note: Values are expressed as mean ± standard deviation. Different lowercase letters within the same column indicate significant differences among treatments at *p* < 0.05 (one-way ANOVA followed by Tukey’s HSD test). The number of samples was *n* = 21 for all treatments (*T0*–*T4*), except *T5*, for which *n* = 28.

**Table 5:** Different metabolomic profiles of the orange fruit peel from the different treatments in the 2024 campaign. Concentrations of metabolites (mM) identified and values are presented as mean (±standard deviation), where the first value represents the average of three replicate measurements, and the value in parentheses represents the standard deviation.

Metabolite	Study Treatment					
	*T0*	*T1*	*T2*	*T3*	*T4*	*T5*
	**Amino acids**					
GABA	0.43 ± 0.08 ^a^	0.56 ± 0.05 ^a^	0.50 ± 0.06 ^a^	0.53 ± 0.04 ^a^	0.47 ± 0.00 ^a^	0.55 ± 0.03 ^a^
Alanine	0.50 ± 0.11 ^a^	0.47 ± 0.01 ^a^	0.46 ± 0.05 ^a^	0.41 ± 0.02 ^a^	0.40 ± 0.01 ^a^	0.40 ± 0.01 ^a^
Asparagine	1.46 ± 0.58 ^a,b^	1.67 ± 0.13 ^b,c^	1.19 ± 0.15 ^b^	2.21 ± 0.21 ^c^	2.23 ± 0.03 ^c^	1.97 ± 0.05 ^a,c^
Aspartate	0.63 ± 0.16 ^a^	0.56 ± 0.13 ^a^	0.59 ± 0.10 ^a^	0.64 ± 0.08 ^a^	0.41 ± 0.02 ^a^	0.51 ± 0.06 ^a^
Glutamate	0.40 ± 0.13 ^a^	0.84 ± 0.17 ^b^	0.77 ± 0.15 ^b,c^	0.61 ± 0.09 ^a,b^	0.52 ± 0.01 ^a,c^	0.72 ± 0.07 ^a,b^
Glutamine	0.24 ± 0.11 ^a^	0.43 ± 0.15 ^a^	0.44 ± 0.08 ^a^	0.41 ± 0.09 ^a^	0.31 ± 0.02 ^a^	0.39 ± 0.06 ^a^
Phenylalanine	0.13 ± 0.01 ^a^	0.11 ± 0.02 ^a,b^	0.13 ± 0.01 ^a^	0.11 ± 0.01 ^a,b^	0.08 ± 0.00 ^b^	0.09 ± 0.00 ^b^
Proline	1.07 ± 0.24 ^a,b^	1.28 ± 0.33 ^b,c^	0.77 ± 0.09 ^b^	1.36 ± 0.25 ^a,c^	1.65 ± 0.09 ^c^	0.87 ± 0.04 ^a,b^
Leucine	0.06 ± 0.00 ^a,b^	0.06 ± 0.00 ^a,c^	0.07 ± 0.01 ^a^	0.06 ± 0.00 ^a,b^	0.05 ± 0.00 ^b^	0.05 ± 0.00 ^b,c^
	**Organic acids**					
Ascorbate	2.00 ± 0.08 ^a^	3.35 ± 0.49 ^b^	3.02 ± 0.29 ^b,c^	2.49 ± 0.26 ^a,c^	2.43 ± 0.07 ^a,c^	2.16 ± 0.22 ^a^
Citrate	0.72 ± 0.08 ^a^	1.07 ± 0.05 ^b,c^	0.98 ± 0.13 ^c,d^	0.92 ± 0.03 ^b,d^	0.80 ± 0.01 ^a,d^	1.14 ± 0.06 ^c^
Formate	0.01 ± 0.00 ^a^	0.01 ± 0.00 ^a^	0.01 ± 0.00 ^a^	0.01 ± 0.00 ^a^	0.01 ± 0.00 ^a^	0.01 ± 0.00 ^a^
Fumarate	0.04 ± 0.00 ^a^	0.03 ± 0.00 ^a^	0.04 ± 0.01 ^a^	0.04 ± 0.00 ^a^	0.03 ± 0.00 ^a^	0.03 ± 0.00 ^a^
Lactate	0.15 ± 0.00 ^a^	0.14 ± 0.01 ^a,b^	0.14 ± 0.01 ^a,b^	0.12 ± 0.01 ^b,c^	0.11 ± 0.01 ^c^	0.11 ± 0.01 ^b,c^
Malate	1.96 ± 0.06 ^a^	2.12 ± 0.03 ^a,b^	2.25 ± 0.22 ^a,c^	2.41 ± 0.24 ^b,c,d^	2.64 ± 0.02 ^d^	2.60 ± 0.05 ^c,d^
Quinic acid	5.64 ± 1.05 ^a^	6.95 ± 0.89 ^a,b^	8.66 ± 0.79 ^b^	6.15 ± 0.36 ^a,c^	5.46 ± 0.31 ^a^	8.00 ± 0.24 ^b,c^
Succinate	0.03 ± 0.00 ^a^	0.04 ± 0.01 ^a^	0.05 ± 0.01 ^a^	0.04 ± 0.01 ^a^	0.03 ± 0.00 ^a^	0.05 ± 0.01 ^a^
	**Sugars**					
Fructose	36.72 ± 0.54 ^a,b^	42.00 ± 1.07 ^b^	41.77 ± 3.24 ^b^	38.65 ± 3.18 ^a,b^	35.15 ± 0.82 ^a^	37.33 ± 0.59 ^a,b^
Glucose	31.72 ± 2.00 ^a,b^	35.04 ± 1.91 ^a,b^	36.68 ± 2.58 ^a^	33.16 ± 2.73 ^a,b^	29.88 ± 0.73 ^b^	30.43 ± 1.41 ^b^
myo-Inositol	2.79 ± 0.09 ^a^	2.60 ± 0.31 ^a,b^	2.45 ± 0.12 ^a,b^	2.36 ± 0.19 ^a,b^	2.42 ± 0.07 ^a,b^	2.24 ± 0.20 ^b^
Sucrose	15.51 ± 0.23 ^a,b^	18.22 ± 0.71 ^c^	15.15 ± 1.27 ^a^	11.71 ± 0.68 ^d^	15.74 ± 0.29 ^a,b^	17.39 ± 0.37 ^b,c^
	**Other metabolites**					
Choline	0.60 ± 0.02 ^a^	0.60 ± 0.04 ^a^	0.62 ± 0.04 ^a^	0.45 ± 0.04 ^b^	0.55 ± 0.02 ^a,c^	0.48 ± 0.01 ^b,c^
Ethanol	0.38 ± 0.01 ^a,b^	0.39 ± 0.01 ^a,b^	0.40 ± 0.05 ^a^	0.37 ± 0.02 ^a,b^	0.33 ± 0.01 ^b^	0.36 ± 0.01 ^a,b^

Note: Values are expressed as mean ± standard deviation. Different lowercase letters within the same row indicate significant differences among treatments at *p* < 0.05 (one-way ANOVA followed by Tukey’s HSD test).

**Table 6:** Different metabolomic profiles of orange juice from the different treatments in the 2024 campaign. Concentrations of metabolites (mM) identified and values are presented as mean (±standard deviation), where the first value represents the average of three replicate measurements, and the value in parentheses represents the standard deviation.

Metabolite	Study Treatment					
	*T0*	*T1*	*T2*	*T3*	*T4*	*T5*
	**Amino acids**					
GABA	0.39 ± 0.06 ^a^	0.36 ± 0.11 ^a^	0.37 ± 0.03 ^a^	0.52 ± 0.14 ^a^	0.37 ± 0.02 ^a^	0.53 ± 0.01 ^a^
Alanine	0.42 ± 0.03 ^a^	0.54 ± 0.06 ^ab^	0.69 ± 0.04 ^b^	0.66 ± 0.12 ^bc^	0.48 ± 0.06 ^ac^	0.65 ± 0.10 ^bc^
Asparagine	6.34 ± 0.56 ^a^	6.89 ± 1.04 ^a^	7.20 ± 1.44 ^ab^	3.42 ± 0.72 ^c^	6.77 ± 0.58 ^a^	9.54 ± 0.18 ^b^
Aspartate	1.67 ± 0.13 ^a^	1.46 ± 0.26 ^ab^	0.83 ± 0.26 ^bc^	1.44 ± 0.35 ^ab^	0.72 ± 0.09 ^c^	0.90 ± 0.25 ^bc^
Arginine	3.67 ± 0.12 ^ab^	3.64 ± 0.60 ^ab^	3.79 ± 0.00 ^a^	3.59 ± 0.42 ^ab^	3.27 ± 0.29 ^ab^	2.70 ± 0.34 ^b^
Glutamate	0.85 ± 0.08 ^ab^	0.87 ± 0.11 ^bc^	1.31 ± 0.28 ^c^	1.21 ± 0.19 ^ac^	0.63 ± 0.11 ^b^	1.07 ± 0.13 ^bc^
Glutamine	0.89 ± 0.06 ^ab^	0.92 ± 0.14 ^bc^	0.89 ± 0.13 ^ab^	1.34 ± 0.32 ^c^	0.73 ± 0.09 ^b^	1.25 ± 0.03 ^ac^
Isoleucine	0.04 ± 0.01 ^ab^	0.02 ± 0.01 ^a^	0.04 ± 0.00 ^ab^	0.03 ± 0.00 ^ab^	0.05 ± 0.01 ^b^	0.04 ± 0.00 ^ab^
Leucine	0.05 ± 0.02 ^a^	0.06 ± 0.04 ^a^	0.04 ± 0.00 ^a^	0.02 ± 0.01 ^a^	0.08 ± 0.01 ^a^	0.06 ± 0.01 ^a^
Proline	2.15 ± 0.15 ^a^	2.51 ± 0.25 ^ab^	3.49 ± 1.23 ^ab^	3.43 ± 0.35 ^ab^	2.41 ± 0.35 ^ab^	3.79 ± 0.17 ^b^
Valine	0.07 ± 0.00 ^a^	0.07 ± 0.02 ^a^	0.08 ± 0.02 ^a^	0.08 ± 0.02 ^a^	0.05 ± 0.01 ^a^	0.09 ± 0.01 ^a^
	**Organic acids**					
Ascorbate	0.60 ± 0.30 ^a^	0.14 ± 0.07 ^ab^	0.04 ± 0.02 ^b^	0.38 ± 0.31 ^ab^	0.25 ± 0.19 ^ab^	0.56 ± 0.11 ^ab^
Citrate	27.75 ± 2.22 ^ab^	27.85 ± 2.01 ^ab^	27.52 ± 3.19 ^ab^	32.01 ± 3.33 ^a^	23.77 ± 1.47 ^b^	27.23 ± 0.95 ^ab^
Malate	10.95 ± 1.13 ^a^	10.85 ± 1.11 ^a^	11.69 ± 1.06 ^a^	10.75 ± 1.71 ^a^	9.20 ± 0.03 ^a^	11.66 ± 0.50 ^a^
Lactate	0.11 ± 0.01 ^a^	0.12 ± 0.00 ^a^	0.16 ± 0.04 ^a^	0.14 ± 0.04 ^a^	0.11 ± 0.00 ^a^	0.14 ± 0.01 ^a^
Succinate	0.02 ± 0.00 ^a^	0.02 ± 0.00 ^a^	0.02 ± 0.01 ^a^	0.04 ± 0.03 ^ab^	0.08 ± 0.01 ^b^	0.02 ± 0.00 ^a^
	**Sugars**					
Fructose	70.41 ± 4.83 ^ab^	64.50 ± 6.74 ^ab^	73.04 ± 3.28 ^b^	73.66 ± 2.08 ^b^	58.86 ± 4.11 ^a^	74.02 ± 4.00 ^b^
Glucose	61.21 ± 3.55 ^ab^	51.23 ± 4.63 ^a^	86.90 ± 10.80 ^c^	68.42 ± 5.31 ^bd^	52.88 ± 4.59 ^ad^	75.52 ± 3.28 ^bc^
myo.Inositol	3.02 ± 0.47 ^ab^	1.26 ± 0.66 ^a^	5.97 ± 2.15 ^c^	5.96 ± 0.49 ^c^	5.31 ± 0.48 ^bc^	7.64 ± 0.70 ^c^
Sucrose	85.03 ± 8.23 ^ab^	80.86 ± 10.18 ^ab^	91.54 ± 4.20 ^b^	87.68 ± 2.85 ^ab^	73.87 ± 4.58 ^a^	92.79 ± 0.76 ^b^
	**Other metabolites**					
Choline	0.15 ± 0.03 ^a^	0.10 ± 0.03 ^a^	0.13 ± 0.05 ^a^	0.18 ± 0.10 ^ab^	0.12 ± 0.01 ^a^	0.31 ± 0.01 ^b^

Note: Values are expressed as mean ± standard deviation. Different lowercase letters within the same row indicate significant differences among treatments at *p* < 0.05 (one-way ANOVA followed by Tukey’s HSD test).

**Table 7:** Total sum by biochemical group of all metabolites detected in the orange peel and juice of all the treatments studied. Note: (p): peel; (j): juice. Aa: amino acids; OA: organic acids; S: sugars; OM: other metabolites.

Metabolite	Study Thesis					
	*T0*	*T1*	*T2*	*T3*	*T4*	*T5*
Amino acids (p)	4.92	5.98	4.92	6.34	6.12	5.55
Organic acids (p)	10.55	13.71	15.15	12.18	11.51	14.10
Sugars (p)	86.74	97.86	96.05	85.88	83.19	87.39
Other metabolites (p)	0.98	0.99	1.02	0.82	0.88	0.84
TOTAL (p)	103.19	118.54	117.14	105.22	101.70	107.88
Amino acids (j)	16.54	17.34	18.73	15.74	15.56	20.62
Organic acids (j)	39.43	38.98	39.43	43.32	33.41	39.61
Sugars (J)	219.67	197.85	257.45	235.72	190.92	249.97
Other metabolites (j)	0.15	0.10	0.13	0.18	0.12	0.31
TOTAL (j)	275.79	254.27	315.74	294.96	240.01	310.51
Juice/Peel Aa	3.36	2.90	3.81	2.48	2.54	3.72
Juice/Peel AO	3.74	2.84	2.60	3.56	2.90	2.81
Juice/Peel S	2.53	2.02	2.68	2.74	2.29	2.86
Juice/Peel OM	0.15	0.10	0.13	0.22	0.14	0.37
Juice/Peel Total	2.67	2.15	2.70	2.80	2.36	2.88

## Data Availability

All data generated and analyzed during this study are included in this published article. Additional information is available from the corresponding author upon request.

## Supplementary Materials

The following supporting information can be downloaded at: https://www.mdpi.com/article/10.3390/plants15030386/s1, Figure S1: Biennial record of maximum, average, and minimum monthly temperatures and accumulated chill hours for the period 2023–2024 in the municipality of Orihuela-Alicante (Spain); Figure S2: Biennial record of maximum, average, and minimum relative atmospheric humidity for the period 2023–2024 in the municipality of Orihuela-Alicante (Spain); Figure S3: Biennial record of wind speed and direction for the period 2023–2024 in the municipality of Orihuela-Alicante (Spain); Figure S4: Polar diagram of the most frequent wind direction for the period 2023–2024 in the municipality of Orihuela-Alicante (Spain); Figure S5: Biennial record of accumulated radiation and received precipitation for the period 2023–2024 in the municipality of Orihuela-Alicante (Spain); Table S1: Climatological parameters recorded in the municipality of Orihuela-Alicante (Spain) during the two-year period 2023–2024.
