# Supplementary material for: Integrative Metabolomic and Physiological Responses of Citrus sinensis to Soil Management in a Semi-Arid Orchard
Source: Plants (Basel). 2026 Jan 27;15(3):386. doi: 10.3390/plants15030386 (PMC12899819; doi:10.3390/plants15030386)
Supplement: Supplementary file 1 [file plants-15-00386-s001.zip › plants-4016731-supplementary.pdf]

INTEGRATIVE METABOLOMIC AND PHYSIOLOGICAL RESPONSES OF *Citrus sinensis* TO SOIL MANAGEMENT IN A SEMI-ARID ORCHARD

SUPPLEMENTARY MATERIAL

1. Explanatory figures on the climate of Orihuela in the two-year period 2023-2024

Figure S1. Biennial record of maximum monthly temperatures, average monthly temperatures, minimum monthly temperatures and accumulated chill hours, for the period 2023-2024 in the municipality of Orihuela-Alicante (Spain)

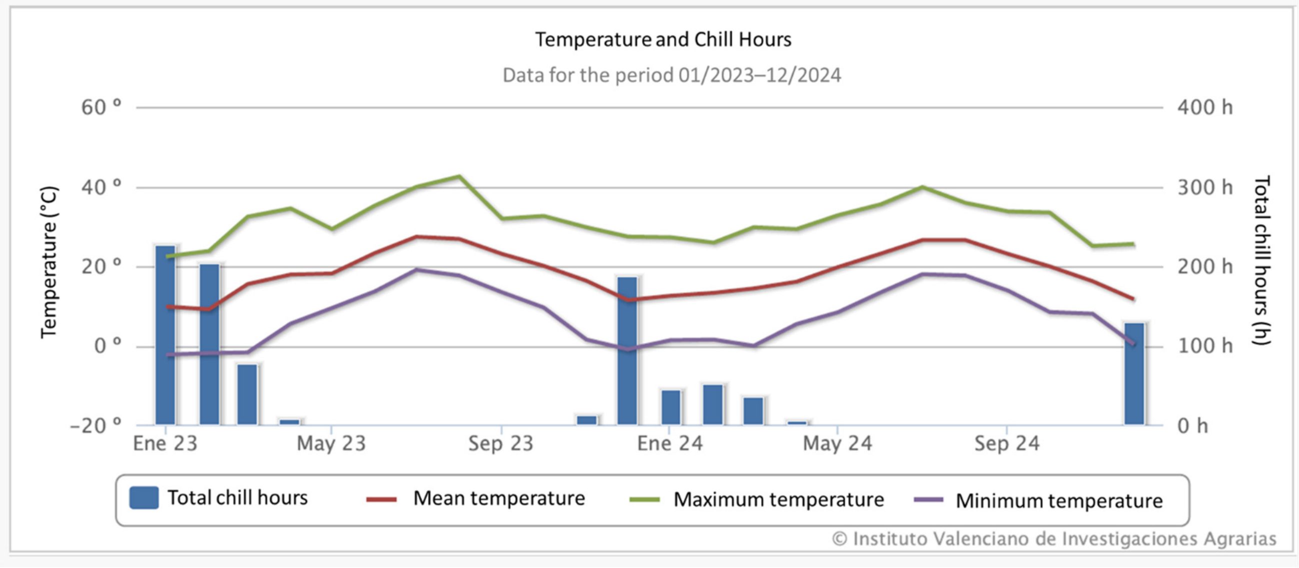

Figure S2. Biennial record of maximum, average and minimum relative atmospheric humidity for the period 2023-2024 in the municipality of Orihuela-Alicante (Spain)

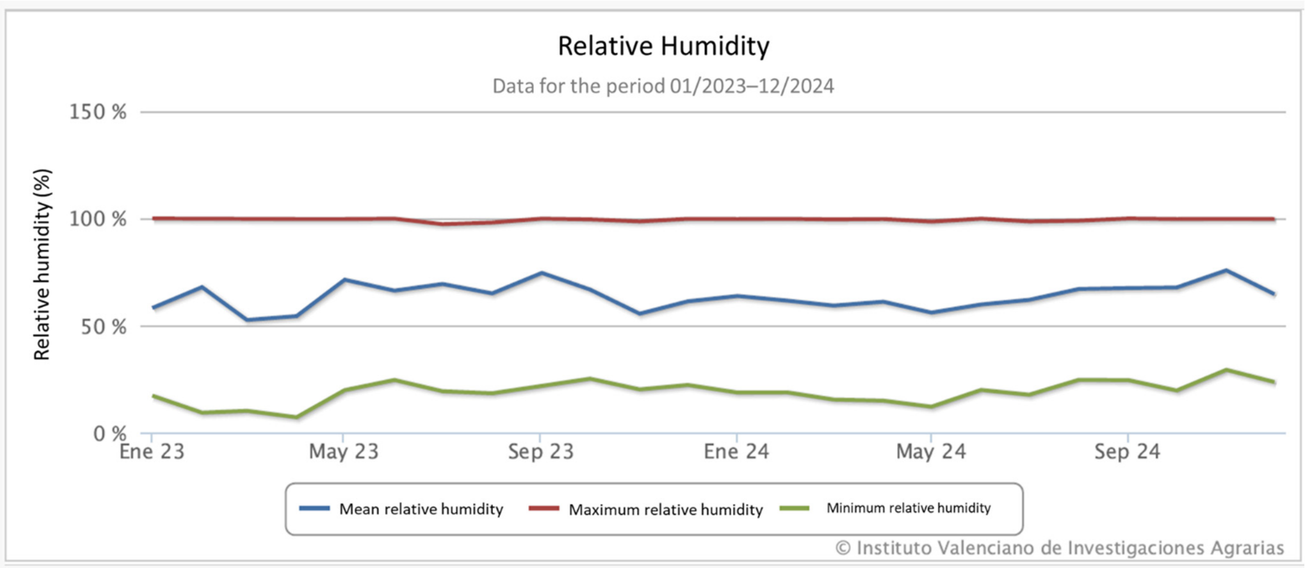

Figure S3. Biennial record of wind speed and direction for the period 2023-2024 in the municipality of Orihuela-Alicante (Spain)

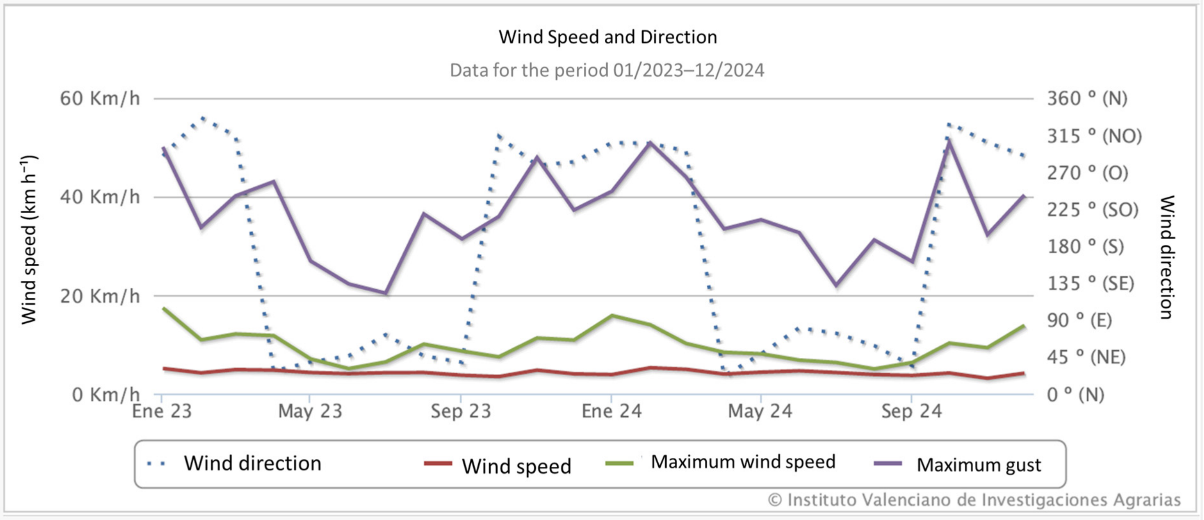

Figure S4. Polar diagram of the most frequent wind direction for the period 2023-2024 in the municipality of Orihuela-Alicante (Spain)

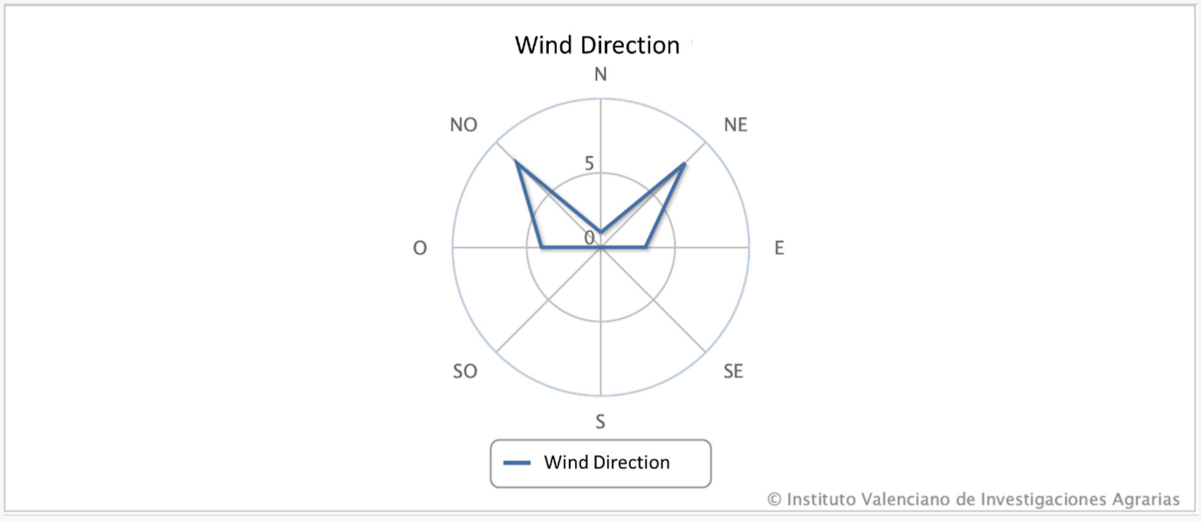

Figure S5. Biennial record of accumulated radiation and received precipitation, for the period 2023-2024 in the municipality of Orihuela-Alicante (Spain)

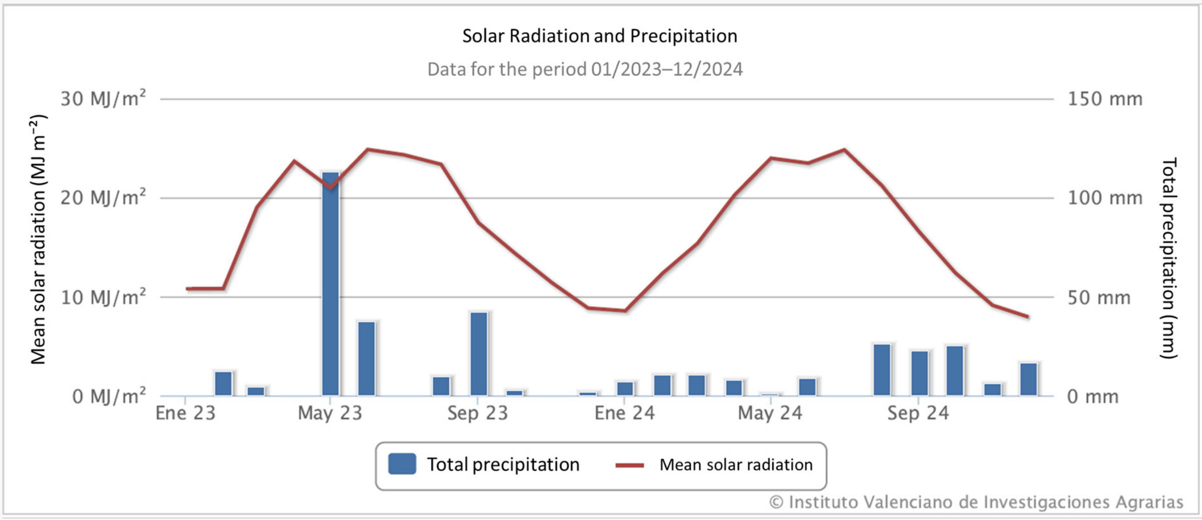

2. Further Information on Experimental Treatments and Clarification of Technical Terms

T0 (Control): No weed barrier, subsurface drainage, or special soil amendments were applied. This treatment represents the traditional cultivation system, serving as the absolute control for the experiment to compare the effects of the implemented treatments.

T1: Weed barrier and subsurface drainage were used, placed between the rows of trees, without any other modifications. The rows are 6 m apart, so the drainage pipe was positioned between the two rows, equidistant from them and 3 m parallel to the crop's centerline. This treatment simulates the conditions of established plantations with mature trees, where soil management and drainage aim to optimize growth in established systems.

T2: Weed barrier and subsurface drainage were used, placed directly beneath the rows of trees, completely parallel to the crop's centerline. No other modifications were made. This treatment simulates new plantings, where drainage is installed beneath the row before planting to promote initial tree establishment and water distribution in the soil.

T3: Weed barrier fabric and subsurface drainage were combined beneath the tree rows, along with a gravel trench. This treatment simulates a traditional drainage system, integrating water management techniques historically used to prevent waterlogging and improve soil aeration.

T4: Weed barrier fabric and subsurface drainage were applied beneath the tree rows, with the addition of zeolite as a soil amendment. This treatment aims to evaluate the effect of natural soil amendments in combination with water management and weed control, promoting fertility and nutrient retention.

T5: Weed barrier fabric was used without subsurface drainage or other additions, representing a traditional mulching system. This treatment allows for the evaluation of weed control effects through mulching without drainage intervention, comparing the results with other, more complex treatments.

NOTE: All pipes were installed at the same depth, with a uniform slope ranging from 0.70m to 0.

3. Record of climatic variables of Orihuela obtained in the two-year period 2023-2024

Table S1. Different climatological parameters recorded in the municipality of Orihuela-Alicante (Spain) during the two-year period 2023-2024

Month	Year	Average max temperature (°C)	Average min temperature (°C)	Average relative humidity (°C)	Average wind speed (km/h)	Average max wind speed (km/h)	Average wind direction	Average radiation (MJ/m²)	Average sunshine hours (U)	Average chill hours (U)	Total precipitation (mm)
1	2023	17,16	3,50	58,20	5,15	17,42	O	10,77	7,68	227,00	0,61
2	2023	15,57	3,17	67,91	4,24	10,92	NO	10,80	8,01	203,50	12,59
3	2023	23,63	7,76	52,56	4,91	12,14	NO	19,01	10,25	76,50	4,87
4	2023	25,72	10,51	54,41	4,80	11,81	NE	23,65	11,37	8,00	0,00
5	2023	24,82	12,96	71,39	4,32	7,07	NE	20,96	10,72	0,00	113,04
6	2023	29,87	17,40	66,29	4,06	5,12	NE	24,85	11,99	0,00	37,52
7	2023	33,85	21,78	69,41	4,29	6,44	E	24,31	12,18	0,00	0,21
8	2023	33,83	20,72	64,97	4,32	10,07	NE	23,35	11,56	0,00	9,84
9	2023	28,99	18,05	74,53	3,77	8,65	NE	17,45	10,05	0,00	43,05
10	2023	27,02	14,19	66,82	3,50	7,48	NO	14,39	9,33	0,00	3,08
11	2023	23,15	10,25	55,54	4,79	11,32	O	11,40	8,36	11,50	0,00
12	2023	18,68	5,12	61,29	4,04	10,88	O	8,83	7,39	186,50	2,05
1	2024	18,72	7,44	63,78	3,89	15,86	NO	8,55	6,90	44,00	7,38
2	2024	20,00	7,41	61,65	5,26	14,01	NO	12,34	8,39	52,00	10,45
3	2024	21,60	7,99	59,32	4,98	10,22	NO	15,37	9,49	35,50	10,66
4	2024	23,43	9,44	61,15	4,01	8,42	N	20,27	10,82	4,50	8,20
5	2024	26,78	12,58	56,03	4,37	8,12	NE	23,97	12,06	0,00	1,03
6	2024	29,52	17,30	59,83	4,66	6,83	E	23,47	12,09	0,00	9,02
7	2024	33,62	20,25	61,99	4,32	6,35	E	24,81	12,35	0,00	0,21
8	2024	33,02	20,90	67,05	3,88	5,04	NE	21,22	11,14	0,00	26,44
9	2024	29,19	17,90	67,49	3,71	6,36	NE	16,56	9,93	0,00	22,75
10	2024	25,69	14,49	67,78	4,20	10,26	NO	12,42	8,75	0,00	25,42
11	2024	21,98	11,44	75,80	3,15	9,34	NO	9,12	7,50	0,00	6,56
12	2024	17,92	5,96	64,62	4,17	13,85	O	7,93	7,03	130,50	16,81
